# Effect of an acute exercise bout on alveolar–capillary membrane permeability and interstitial fluid accumulation in COPD

**DOI:** 10.1113/EP094018

**Published:** 2026-09-04

**Authors:** Rie Skovly Thomsen, Iben Elmerdahl Rasmussen, Stine Buus Nymand, Anna Agnes Lytzen, Jacob Peter Hartmann, Milan Mohammad, Malte Lund Adamsen, Birgitte Hanel, Jann Mortensen, Ronan M. G. Berg

**Affiliations:** ^1^ Centre for Physical Activity Research Copenhagen University Hospital – Rigshospitalet Copenhagen Denmark; ^2^ Department of Clinical Physiology and Nuclear Medicine Copenhagen University Hospital – Rigshospitalet Copenhagen Denmark; ^3^ Department of Biomedical Sciences, Faculty of Health and Medical Sciences University of Copenhagen Copenhagen Denmark; ^4^ Department of Cellular and Molecular Medicine University of Copenhagen Copenhagen Denmark; ^5^ COPEACT – Copenhagen Research Centre for Autoimmune Connective Tissue Disease Copenhagen University Hospital – Rigshospitalet Copenhagen Denmark; ^6^ Department of Clinical Medicine University of Copenhagen Copenhagen Denmark; ^7^ Department of Medicine The National Hospital Torshavn Faroe Islands; ^8^ Faculty of Health Sciences University of the Faroe Islands Torshavn Faroe Islands; ^9^ Neurovascular Research Laboratory, Faculty of Life Sciences and Education University of South Wales Pontypridd UK

**Keywords:** alveolar–capillary membrane breaching, diffusing capacity, dyspnoea, interstitial oedema, stress failure, NCT06287476

## Abstract

Exertional dyspnoea is a debilitating symptom in chronic obstructive pulmonary disease (COPD) and often persists after the cessation of exercise. The underlying mechanisms are not fully understood, and a potential contributing factor that has not previously been investigated is alveolar–capillary membrane breaching with extravasation of fluid into the lungs. Sixteen individuals with COPD and 16 age‐ and sex‐matched healthy controls were included. Alveolar–capillary membrane permeability was quantified using the pulmonary clearance index (PCI), calculated from scintigraphically determined alveolar clearance of ^99m^Tc‐labelled diethylenetriaminepentaacetic acid at rest and following an acute exercise bout performed at maximal exertion, as determined by a prior cardiopulmonary exercise test. In addition, lung tissue mass (LTM) was assessed using low‐dose computed tomography, with rest‐to‐post‐exercise changes interpreted as reflecting alterations in interstitial fluid accumulation. The mean change in PCI from rest‐to‐post‐exercise was −0.01 [95% CI: −0.09, 0.06]%/min in the COPD group and −0.05 [95% CI: −0.12, 0.02]%/min in the healthy control group (Group × Time interaction, *P* = 0.446). The mean change in LTM from rest‐to‐post‐exercise was 16.5 [95% CI: −7.0, 40.0]g/1.73 m^2^ in the COPD group and 35.2 [95% CI: 11.7, 58.7]g/1.73 m^2^ in the healthy control group (Group × Time interaction, *P* = 0.258). In conclusion, the present study found no evidence of alveolar–capillary membrane breaching following maximal exercise, neither in individuals with COPD nor in healthy matched controls.

## Abstract (highlights)

- **What is the central question of this study?**
  Is exercise at maximal exertion associated with alveolar–capillary membrane breaching and interstitial fluid accumulation in the lungs of individuals with COPD?
- **What is the main finding and its importance?**
  Alveolar–capillary membrane permeability and lung tissue mass were unaffected by an acute exercise bout at maximal exertion, both in individuals with COPD and in age‐ and sex‐matched healthy controls. Thus, alveolar–capillary membrane breaching with concomitant fluid extravasation into the lungs is unlikely to contribute to persistent post‐exercise dyspnoea in either of these groups.

## 1 INTRODUCTION

Individuals with chronic obstructive pulmonary disease (COPD) experience a downward spiral characterised by progressive dyspnoea, physical inactivity and deconditioning, which in turn exacerbate dyspnoea, leading to poor quality of life, disability and, in many cases, premature death (Gagnon et al., 2014; GOLD, 2024). As part of this marked reduction in exercise capacity, exertional dyspnoea is a common limiting factor (Van Remoortel et al., 2013). The proposed mechanisms include dynamic hyperinflation, although the aetiology is multifactorial and not yet fully understood (D'Cruz et al., 2026; Guenette et al., 2012). Consequently, dyspnoea often persists after the cessation of exercise, even after dynamic hyperinflation has resolved (Marques‐Magallanes et al., 1998; Stevenson & Calverley, 2004).

A hitherto largely unexplored mechanism that may contribute to exertional dyspnoea persisting beyond acute exercise in COPD is interstitial fluid accumulation. This phenomenon is known to cause dyspnoea through activation of vagal afferents by irritant J‐receptors located in the alveolar septae, as described in other conditions (Burki & Lee, 2010; Fukushi et al., 2021) including heart failure and following exercise to maximal exertion in elite athletes (Burki & Lee, 2010; Hårdstedt et al., 2021; Luks et al., 2007). Although previous studies indicate that alveolar–capillary membrane permeability is generally normal in non‐smoking individuals with COPD (Huchon et al., 1984; Rinderknecht et al., 1980; Sundram, 1995), exercise‐induced interstitial fluid accumulation may nevertheless arise from transient breaches in the alveolar–capillary membrane secondary to increased lung inflation and elevated pulmonary vascular pressures (Egan, 1980; Herrero et al., 2018; Marks et al., 1985). This has previously been described primarily in the context of maximal exertion in elite athletes who achieve exceedingly high cardiac outputs and pulmonary vascular pressures (Hanel et al., 2003; Hopkins et al., 1997; Lorino et al., 1989; Meignan et al., 1986), but a similar phenomenon may occur in individuals with COPD. Thus, consistent with the well‐documented reduction in alveolar–capillary reserve, with a diminished capacity for pulmonary capillary recruitment and distension during exercise in COPD (Behnia et al., 2017; Hartmann et al., 2025; Tedjasaputra et al., 2018), relatively large exercise‐induced increases in pulmonary vascular pressures occur at a given cardiac output compared with the healthy state (Hilde et al., 2013). Also, the presence of chronic airway inflammation may concomitantly render the pulmonary microvasculature more vulnerable to the mechanical stress imposed by exercise‐induced increases in pulmonary vascular pressures (Herrero et al., 2018; Kyomoto et al., 2019).

Therefore, in the present study, we compared the impact of an acute exercise bout performed at maximal exertion on alveolar–capillary membrane permeability and lung tissue mass (LTM) in individuals with COPD to that of age‐ and sex‐matched healthy controls. We hypothesised that maximal exercise would increase alveolar–capillary membrane permeability in COPD compared to healthy controls, indicative of alveolar–capillary membrane breaching, with a concomitantly greater increase in LTM due to fluid extravasation within the lung.

## 2 METHODS

### 2.1 Ethical approval

This project was approved by the Regional Ethical Committee of the Capital Region of Denmark (file no. H‐23043870), and the study was registered at ClinicalTrials.gov (ID NCT06287476). The study was performed according to the most recent guidelines of the *Declaration of Helsinki*. All participants provided oral and written informed consent prior to enrolment.

### 2.2 Study design and setting

This study is reported according to the ‘Strengthening the reporting of observational studies in epidemiology (STROBE) statement: guidelines for reporting observational studies’ (von Elm et al., 2007). A total of 32 participants, 16 individuals with COPD and 16 healthy age‐ and sex‐matched controls were recruited to participate in the study. The study was conducted at the Centre for Physical Activity Research and Department of Clinical Physiology and Nuclear Medicine both at Rigshospitalet, Copenhagen, Denmark.

Each subject completed three study days. Baseline measurements included a medical health examination, standardised lung function testing, and a cardiopulmonary exercise test (CPET). On the second study day, participants underwent low‐dose computed tomography (LDCT) scans before and after exercise, followed by measurement of the pulmonary clearance index (PCI) using ^99m^Tc‐labelled diethylenetriaminepentaacetic acid (^99m^Tc‐DTPA); this was done at rest and post‐exercise when the breathing pattern had normalised; to ensure that the timing did not differ between groups, this was pragmatically set a priori at a minimum of 10 min. On the third study day, pulmonary diffusing capacity (*D*_L,CO,NO_) was assessed using the single‐breath dual test‐gas method with carbon monoxide (CO) and nitric oxide (NO). Measurements of *D*_L,CO,NO_ were performed in the supine position both at rest and post‐exercise; the timing of the post‐exercise measurement was matched with that performed on the second study day (Appendix Figure A1). The study days were separated by a minimum of 48 h. Before each visit participants were instructed to abstain from caffeine and alcohol for 16 h, nicotine for 10 h, and vigorous physical activity for 48 h.

### 2.3 Participants

The study included participants diagnosed with mild to severe COPD classified as stage I to III according to the Global Initiative for Chronic Obstructive Lung disease (GOLD) guidelines (GOLD, 2024). For comparison, healthy age‐ and sex‐matched controls (matched 1:1) were included.

Inclusion criteria for COPD participants consisted of (1) individuals of both sexes, (2) age between 45 and 80 years, (3) COPD GOLD stage I to III, and (4) resting arterial oxygenation above 90%. For the healthy controls the inclusion criteria consisted of (1) individuals of both sexes, (2) age 45–80 years, and (3) normal lung function including dynamic and static lung volumes and diffusing capacity for CO adjusted for haemoglobin with a 10‐s breath‐hold (*D*_L,COc,10s_). Exclusion criteria for both groups consisted of symptoms of ischaemic heart disease or arrythmias, known heart failure, symptoms of illness within 2 weeks prior to the study, pregnancy, known renal or liver disease, and active smoking. Furthermore, exclusion criteria for healthy controls were known chronic lung disease.

### 2.4 Experimental set‐up

#### 2.4.1 Medical examination

The medical examination included auscultation of the heart and lungs, a medical history review, electrocardiogram, blood pressure, heart rate and all current drug use to confirm the absence of any exclusion criteria. Height (m) and weight (kg) were measured, and body mass index (BMI, kg/m^2^) was calculated (weight in kg/(height in m)^2^).

#### 2.4.2 Lung function testing

All lung function tests were performed in the upright seated position using the Jaeger MasterScreen PFT pro system (CareFusion, Höchberg, Germany), and included dynamic spirometry, whole‐body plethysmography and single‐breath uptake of CO. All tests were performed by trained personnel (Bhakta et al., 2023; Graham et al., 2019). Data obtained from lung function testing included: forced expiratory volume in 1 s (FEV_1_), forced vital capacity (FVC), FEV_1_/FVC ratio, total lung capacity (TLC), residual volume (RV) and *D*_L,COc,10s_, both as absolute values and as percent of predicted values according to standard reference equations (Cotes et al., 1993; Quanjer et al., 1993; Stanojevic et al., 2022). Prior to measurements, participants' haemoglobin (Hb) levels were obtained. Hb measurements were performed using capillary blood samples and analysed with the HemoCue device (Hb 201+; HemoCue AB, Ängelholm, Sweden). Given that the low‐dose CT scans obtained for determining LTM (see below) were not suitable for identifying or quantifying emphysema, individuals with evidence of static hyperinflation (RV and/or RV/TLC above the upper limit of normal) together with a *D*_L,COc,10s_ below the lower limit of normal were operationally classified as having a physiological phenotype consistent with emphysema (Dubé et al., 2016).

#### 2.4.3 CPET

The peak oxygen consumption test was completed on a bicycle ergometer (LC4 or LC6, Monark Exercise AB, Vansbro, Sweden). The test commenced with a 5‐min baseline measurement seated at rest on the bike followed by a 5‐min warm‐up. This was followed by an incremental increase in workload every minute until exhaustion. The test was terminated either due to medical conditions, reaching less than 60 rpm for 20 s, saturation level dropping below 80%, or voluntarily cessation by the participant. Workload and increments were calculated for the individual participant in accordance with physical activity level, age, sex, waist measurement and resting heart rate (Nes et al., 2011). Respiratory gases and minute ventilation were measured using a Quark gas analyser (COSMED Quark CPET system, COSMED srl, Rome Italy) using breath‐by‐breath method. Heart rate was measured with a smartLAB device (Dossenheim, Germany) chest band compatible with COSMED system. Saturation was measured by pulse oximetry (Nonin, Xpod). Both an objective assessment of exhaustion, including (1) respiratory exchange ratio (RER) > 1.1, (2) Borg scale > 17, and (3) plateau in ${\dot{V}}_{O_{2}}$ (Basset & Howley, 2000; Radtke et al., 2019), and a subjective assessment of exhaustion evaluated by the operator were conducted following test termination. The highest reached oxygen uptake (${\dot{V}}_{O_{2}}$) was reported as peak oxygen uptake (${\dot{V}}_{O_{2}\mathrm{peak}}$). Rate of perceived exhaustion was measured using the Borg scale (6–20) at test termination rating overall exhaustion, lung and leg exhaustion (Borg, 1982). Additionally, a peak tidal volume (*V*_Tpeak_)/TLC ratio of ≤0.22 was used as to identify the presence of airflow obstruction with dynamic hyperinflation (Chuang, 2023).

#### 2.4.4 Acute exercise bout

The exercise protocol on both study day 2 and 3 commenced with a 5‐min warm‐up period at 30% of peak workload (*W*_L,peak_) determined from the CPET from study day 1, followed by an acute exercise bout consisting of three intervals: interval (1) 4 min at 60% *W*_L,peak,_ interval (2) 4 min, also at 60% *W*_L,peak_ and interval (3) until exhaustion or for a maximum of 4 min at 100% *W*_L,peak_. The three intervals were separated by a 2 min active break at 15 W.

#### 2.4.5 Alveolar–capillary membrane permeability

At rest and post‐exercise, alveolar–capillary membrane permeability was quantified by PCI, calculated from the alveolar clearance of ^99m^Tc‐DTPA (Groth, 1991). Diethylenetriaminepentaacetic (DTPA) is a hydrophilic polar molecule, and its clearance from the alveolar space into the pulmonary capillary blood occurs by diffusion through tight junctions, with transport across the alveolar epithelium constituting the rate limiting step, because its permeability to DTPA is approximately 10‐fold lower than that of the capillary endothelium (Rinderknecht et al., 1980). This is because DTPA has a molecular radius of approximately 0.6 nm and is therefore able to diffuse freely through the pores of the tight junctions of the alveolar epithelium, which have a radius of approximately 0.8–1.0 nm; the corresponding pore radius in the endothelial tight junctions is 4.0–8.0 nm (Taylor & Gaar, 1970).

In the supine position, participants inhaled ^99m^Tc‐DTPA aerosol for 5 min. In accordance with established methodology, the same protocol was applied as previously described (Hanel et al., 2003). Briefly, the aerosol was generated from 400 MBq (baseline inhalation) and 800 MBq (post‐exercise inhalation) of ^99m^Tc‐DTPA in 5 mL of 0.9% sodium chloride, using a jet nebuliser at a flow rate of 10 L O_2_ per minute (Venti‐Scan IV Convenience Kit, 12 tubing, Mirion Technologies (Capintec), Florham Park, NJ, USA). Pulmonary clearance of DTPA was measured by detecting the decay of ^99m^Tc over the chest in the anterior–posterior plane using a gamma camera equipped with a low‐energy all‐purpose collimator. The energy window was set at 140 keV with a 20% window setting. Data were acquired as a 20‐min dynamic acquisition in 30 s frames with a matrix size of 128 × 128 pixels. For each measurement, all 30 s frames were integrated into a single static image to enable drawing of regions of interest for calculation of PCI.

A washout analysis was conducted using IntelliSpace together with NM‐Lung (IntelliSpace Portal 10.3, Philips Medical Systems, Best, The Netherlands). Regions of interest were drawn for the left and right lungs, including both whole lung and peripheral lung regions, to ensure sufficient alveolar clearance. One region of interest was placed outside the lung to correct for background activity, and a correction for physical decay of technetium was applied. A time–activity curve was generated, from which PCI, defined as the measured rate constant of alveolar ^99m^Tc‐DTPA clearance, was determined as the percentage decrease in activity per minute (%/min). Two time–activity curves, one at rest and one following exercise, were generated for each participant.

#### 2.4.6 LTM

Immediately prior to each alveolar ^99m^Tc‐DTPA clearance measurement, a LDCT (120 kV, 22 mAs quality reference with CARE Dose modulation) was performed in the supine position at functional residual capacity both at rest and following the acute exercise bout. From the LDCT, LTM was calculated using an identical approach to that described in detail elsewhere (Hartmann et al., 2025). The LDCT data were subsequently analysed using HERMES Hybrid 3D, Lung Lobe Quantification v. 3.0 (HERMES Medical Solutions AB, Stockholm, Sweden), together with a customised Python script. A predetermined threshold in Hounsfield units (HU) was applied to delineate lung tissue and to distinguish between airways, vessels and lung parenchyma, with airway thresholds set between −1000 and −900 HU and lung tissue thresholds between −800 and −300 HU for the different predefined presets. The same preset was used at rest and post‐exercise within each individual participant if possible. Data were transferred to Python 3.9 in which a script was used to calculate density (ρ) in g/ml from the HU assuming a direct linear relationship between voxel attenuation and physical density $\rho =\frac{\mathrm{HU}}{1000}+1$. Mass was then calculated by multiplying the density with the corresponding volume, yielding an estimate of total LTM that excludes the airways and pulmonary vasculature except from the microvasculature. LTM was reported both as absolute values and normalised to 1.73 m^2^ body surface area using the DuBois formula (Du Bois & Du Bois, 1916)

#### 2.4.7 D_L,CO,NO_

Measurement of *D*_L,CO,NO_ was conducted before and post‐exercise by the single‐breath manoeuvre with a 5‐s breath‐hold as described in detail elsewhere (Nymand et al., 2024), to provide the diffusing capacity for NO (*D*_L,NO_) and for CO (*D*_L,CO,5s_). To permit comparison with conditions during LDCT, all measurements were conducted in the supine position, both at rest and post‐exercise. For each participant, the timing of the post‐exercise *D*_L,CO,NO_ measurements were matched to the timing of LDCT acquisition using a stopwatch.

Participants were equipped with a nose‐clip and initiated normal tidal breathing through a mouthpiece (SpiroBac, Henrotech, Aartselaar, Belgium) (dead space 56 mL, resistance to flow 12 L s^−1^ 0.9 cmH_2_O) after automatic resetting of the device. Participants were instructed to exhale to RV, followed by a rapid inspiration to TLC in which the valve opened and the gases inhaled (0.28% CO, 9.3% He, 20.9% O_2_ and 69.52% N_2_). A breath‐hold of 5 s was conducted followed by a rapid expiration. Measurements were repeated after a 4‐min wash‐out period with a minimum of two manoeuvres in each position fulfilling acceptability and repeatability criteria as described elsewhere (Nymand et al., 2024; Zavorsky et al., 2017).

The alveolar–capillary membrane diffusing capacity for CO (*D*_M,CO_) and pulmonary capillary blood volume (*V*_C_) were calculated based on *D*_L,NO_ and *D*_L,CO,5s_ (Thomsen et al., 2025):

$$\;D_{M,\mathrm{CO}}=\frac{\frac{1}{\alpha }-\frac{1}{k}}{\frac{1}{D_{L,\mathrm{NO}}}-\frac{1}{k\times D_{L,\mathrm{CO},5s}}}V_{C}=\frac{\left(\frac{1}{\theta_{\mathrm{CO}}}\right)\left(1-\frac{\alpha }{k}\right)}{\frac{1}{D_{L,\mathrm{CO},5s}}-\frac{\alpha }{D_{L,\mathrm{NO}}}}$$

α was related to the *D*_M_ ratio $\left(\frac{D_{M,\mathrm{NO}}}{D_{M,\mathrm{CO}}}\right)$ and was set at 1.97, *k* was calculated as the ratio of specific conductances of the two test gases in blood ($\frac{\theta_{\mathrm{NO}}}{\theta_{\mathrm{CO}}})$ with $\theta_{\mathrm{NO}}$ set at 1.51 $mL_{\mathrm{blood}}\;\mathrm{mi}n^{-1\;}\mathrm{kP}a^{-1}\;\mathrm{mmo}{l_{\mathrm{NO}}}^{-1}$ while $\theta_{\mathrm{CO}}$ calculated as described elsewhere (Hartmann et al., 2025). Assuming a $\theta_{O_{2}}$ of 0.6 mmol O_2_ min^−1^ kPa^−1^ mL blood^−1^ corrected for Hb ($\theta_{O_{2},\mathrm{adj}}$ ):

$$\theta_{O_{2},\mathrm{adj}}=0.6\;\mathrm{mmol}\;O_{2}\;\mathrm{mi}n^{-1}\mathrm{kP}a^{-1}\mathrm{mL}\;\mathrm{bloo}d^{-1}\times \left(\frac{\left[\mathrm{Hb}\right]}{9.3\;\mathrm{mM}}\right)$$

The estimated pulmonary diffusing capacity for O_2_ (e*D*_L,O2_) was calculated (Nymand et al., 2026):

$$eD_{L,O_{2}}=\frac{0.738\;\times \;D_{M,\mathrm{CO}}\times \;V_{c}}{\theta_{O_{2},\mathrm{adj}}\times V_{c}+1.23\times D_{M,\mathrm{CO}}}$$

### 2.5 Outcomes

The primary outcome was the rest‐to‐post‐exercise change in PCI, while the associated change in LTM was the secondary outcome. Because LDCT‐based estimates of LTM include the pulmonary capillary bed, *D*_L,CO,NO_‐derived *V*_C_ obtained in the supine position was assessed to account for the potentially confounding influence of this when using rest‐to‐post‐exercise changes in LTM to evaluate the extent of interstitial fluid accumulation.

### 2.6 Potential sources of bias

COPD diagnosis was validated through comprehensive lung function testing aligning with accepted consensus guidelines and evaluated by a physician; thus, the likelihood of COPD misclassification was minimal. All measurements were performed according to standardised guidelines with trained personnel. Therefore, the bias is expected to be low. However, since mainly healthier individuals with COPD signed up, selection bias may occur in this study.

### 2.7 Sample size

SD of DTPA PCI was expected to be around 1.0%/min in both groups according to previous data (Hanel et al., 2003). For detection of a between‐group difference of 0.45%/min with a statistical power of 90% and a two‐sided significance level of 0.05, *n* = 16 was thus required in each group.

### 2.8 Statistical methods

All statistical analyses were conducted using R statistical software version 4.3.0 (R Foundation for Statistical Computing, Vienna, Austria) within RStudio (version 1.4.1717). Normality of the data was assessed using visual inspection by histograms. Normally distributed variables are reported as mean (SD) and mean difference (95% CI: lower limit (LL), upper limit (UL)); otherwise, non‐normally distributed data are reported as median [25th percentile–75th percentile]. Student's *t*‐test was used to detect differences in baseline characteristics between groups and a Mann–Whitney *U*‐test was used when assumption of normality failed. A similar statistical approach was applied to examine between‐group and within‐group differences from the acute exercise bouts.

A linear mixed effect model was conducted using the lme4 package (lme4 package; version 1.1‐35.3). ID was set as random effect to account for repeated measurements. Fixed effects included Group (COPD/control), Time (rest/exercise), height and sex. The model specifications were as follow: outcome ∼ Group × Time + Sex + Height + (1 | ID). Additionally, statistically analysis was made including RV as a fixed effect in the model. Model assumptions were assessed via visual inspection of fitted values vs. residual plots and gave no reason of concern. *Post hoc* pairwise comparisons of estimated marginal means were performed to determine mean differences within groups, and Tukey's correction was applied to account for multiple testing. Statistical significance level was set to α < 0.05, two‐sided.

## 3 RESULTS

### 3.1 Participant characteristics

Participant characteristics including lung function and CPET results are presented in Table 1. Groups were similar according to age, height, weight, body mass index and ${\dot{V}}_{O_{2}\mathrm{peak}}$. Individuals with COPD had lower FEV_1_, %FEV_1_, %FVC, FEV_1_/FVC ratio, RV, %*D*_L,COc,10s_, higher lung volumes, higher everyday dyspnoea score measured by The Medical Research Council Dyspnoea Scale (1–5) and more smoking pack years compared to healthy controls. In the COPD group, disease severity was distributed as follows: three with mild COPD (19%), 11 with moderate COPD (69%) and two with severe COPD (12%). Of these three participants in the moderate group and two participants in the severe group had a physiological phenotype consistent with emphysema.

**TABLE 1:** Participant characteristics.

	COPD	Healthy controls	*P*
*n*	16	16	
Sex (F/M)	8/8	8/8	—
Age (years)	68.0 (5.6)	67.0 (4.6)	0.585
Height (cm)	170.4 (13.3)	170.7 (7.6)	0.951
Weight (kg)	78.2 (16.8)	76.8 (15.8)	0.815
BMI (kg/m^2^)	27.0 (5.6)	26.2 (4.4)	0.670
BSA (m^2^)	1.90 (0.24)	1.90 (0.21)	0.927
Smoking (pack years)	40 [30–50]	7 [0–10]	0.001
MRC (1–5)	2 [1–2]	1 [1–1]	0.019
**Lung function**			
FEV_1_ (L)	1.83 (0.79)	3.20 (0.77)	<0.001
FEV_1_ (% predicted)	63.9 (13.8)	112.2 (14.7)	<0.001
FVC (L)	3.49 (1.36)	4.17 (1.02)	0.122
FVC (% predicted)	95.9 (15.0)	113.6 (13.2)	0.001
FEV_1_/FVC	0.51 (0.09)	0.77 (0.06)	<0.001
FEV_1_/FVC (% predicted)	66.6 (11.7)	98.6 (7.0)	<0.001
TLC (L)	6.86 (1.93)	6.50 (1.29)	0.536
TLC (% predicted)	112.8 (12.1)	107.0 (8.8)	0.136
RV (L)	3.17 (1.03)	2.38 (0.35)	0.010
RV (% predicted)	135.8 (31.8)	103.5 (8.9)	0.001
*D*_L, COc, 10s_ (mmol/(min kPa))	6.45 (2.37)	8.04 (2.43)	0.072
*D*_L, COc, 10s_ (% predicted)	76.8 (21.9)	94.9 (16.1)	0.012
IC (L)	2.82 (0.95)	3.21 (0.95)	0.278
Lung tissue mass (g)	622.0 (198.9)	569.8 (95.8)	0.354
Lung tissue mass (g/1.73 m^2^)	561.3 (143.8)	524.6 (82.2)	0.385
CT‐based functional residual capacity (mL)	3572 (1275)	2636 (551)	0.014
**Cardiopulmonary exercise test**			
${\dot{V}}_{O_{2}\mathrm{peak}}$ (mL/min)	1983.1 (729.6)	2376.0 (832.8)	0.166
${\dot{V}}_{O_{2}\mathrm{peak}}$ (mL/min/kg)	25.3 (7.9)	31.3 (9.9)	0.070
HR_peak_ (beats/min)	143.9 (14.1)	155.3 (12.1)	0.021
*W*_L,peak_ (W)	157.3 (79.5)	210.4 (87.5)	0.083
RER_peak_	1.07 (0.12)	1.17 (0.08)	0.010
BORG (6–20)	17.1 (1.8)	17.5 (1.9)	0.565
MVV (L)	73.5 (31.2)	126.7 (30.7)	<0.001
Ventilatory reserve (%)	9.8 (36.9)	28.0 (19.8)	0.095
${\dot{V}}_{\mathrm{Epeak}}$ (L/min)	72.0 (28.3)	97.3 (33.3)	0.028
*V*_Tpeak_ (L)	1.9 (0.7)	2.3 (0.5)	0.062
RF_peak_ (frequency)	38.4 (6.7)	42.1 (8.1)	0.171
${\dot{V}}_{O_{2}}$/HR_peak_	14.9 (5.4)	16.1 (4.9)	0.492
${P_{\mathrm{ET}O_{2}}}_{\mathrm{peak}}$	114.4 (4.4)	118.8 (4.8)	0.010
${P_{\mathrm{ETC}O_{2}}}_{\mathrm{peak}}$	36.0 (4.6)	37.8 (16.5)	0.672

*Note*: Participant characteristics of individuals with chronic obstructive pulmonary disease (COPD) and healthy controls. Data are represented as means (SD); otherwise, median [interquartile range]. Student's *t*‐test was used to detect differences between groups and a Mann–Whitney *U*‐test was used when assumption of normality failed. Estimated maximal voluntary ventilation (MVV) was determined as forced expiratory volume in 1 s (FEV_1_) multiplied by 40.

Abbreviations: *D*_L,COc_, pulmonary diffusing capacity corrected for haemoglobin; FVC, forced vital capacity; HR_peak_, peak heart rate; IC, inspiratory capacity; ${P_{\mathrm{ETC}O_{2}}}_{\mathrm{peak}}$, peak end‐tidal carbon dioxide tension; ${P_{\mathrm{ET}O_{2}}}_{\mathrm{peak}}$, peak end‐tidal oxygen tension; RER_peak_, peak respiratory exchange ratio; RF_peak_, peak respiratory frequency; ${\dot{V}}_{\mathrm{Epeak}}$, peak minute ventilation; ${\dot{V}}_{O_{2}}$/HR_peak_, peak oxygen pulse; ${\dot{V}}_{O_{2}\mathrm{peak}}$, peak oxygen uptake; *V*_Tpeak_, peak tidal volume; *W*_Lpeak_, peak workload.

### 3.2 Acute exercise bout

All participants completed the three exercise intervals at both study days, with no difference in exercise time in the last interval between the two groups (Day 1, COPD: 140 [108–179] s; healthy controls: 122 [91–139] s; *P* = 0.386; Day 2, COPD: 130 [110–219] s; healthy controls: 116 [76–182] s; *P* = 0.192). This was also found between the two study days within the group (COPD: *P* = 0.509; healthy controls: *P* = 0.778), as was peak heart rate (HR_peak_) between the groups on each study day (Day 1: *P* = 0.180; Day 2: *P* = 0.800) and between the two study days (COPD: *P* = 0.969; healthy controls: *P* = 0.321). No differences were found for HR_peak_ comparing HR_peak_ from the CPET and from the last interval in the acute exercise bout (COPD, *P* = 0.518; healthy controls, *P* = 0.716). The COPD group reached 102% of HR_peak_ in the last interval and the healthy control group reached 99% of HR_peak_ compared to the HR_peak_ achieved during the CPET at study day 1. A *V*_Tpeak_/TLC ratio ≤ 0.22 was observed in three participants in the COPD group and in none of the healthy controls.

### 3.3 Alveolar–capillary membrane permeability

Post‐exercise pulmonary ^99m^Tc‐DTPA clearance measurements were conducted 1350 (143) s, and 1332 (136) s post the acute exercise bout in the COPD and healthy control group, respectively (between‐group *P* = 0.616). PCI was similar between groups at rest (COPD: 1.01 [0.86, 1.17]%/min; healthy controls: 0.81 [0.65, 0.97]%/min, (*P* = 0.275)). The mean difference in PCI from rest‐to‐post‐exercise was −0.01 [−0.09, 0.06]%/min (*P* = 0.976) in the COPD group and −0.05 [−0.12, 0.02]%/min (*P* = 0.447) in the healthy control group. No significant effect of RV on PCI was found (data not shown). There was no significant Group × Time interaction. Data on PCI are presented in Figure 1.

**FIGURE 1:**
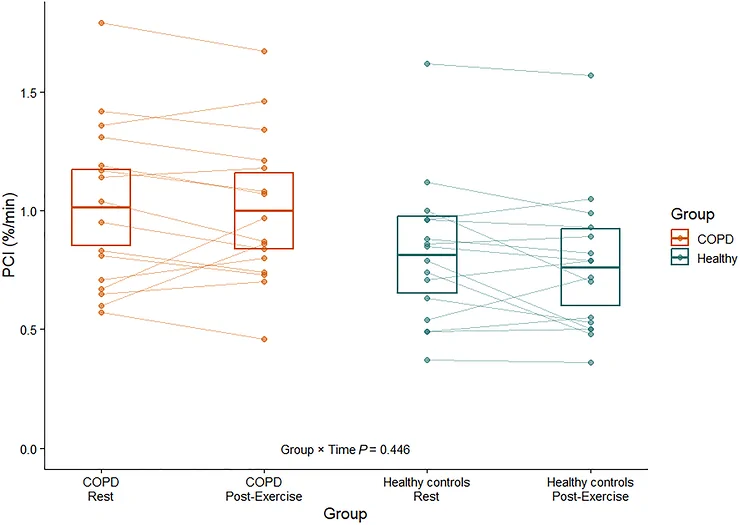
Rest‐to‐post‐exercise changes in pulmonary clearance index. Note: Rest‐to‐post‐exercise changes in pulmonary clearance index (PCI) in COPD participants (orange, *n* = 16) and healthy controls (green, *n* = 16). Each dot represents an estimate of a participant's PCI. The boxes show the estimated marginal means (95% confidence interval) averaged over sex. The overall interaction term reflects the Group ∙ Time interaction effect. COPD, chronic obstructive pulmonary disease.

### 3.4 LTM

Post‐exercise LTM was measured 795 (mean, SD 151) s and 741 (mean, SD 130) s after the acute exercise bout in the COPD and healthy control group, respectively (between‐group *P* = 0.284). LTM at rest was similar between groups (COPD: 564 [510, 617] g/1.73 m^2^; 526 [472, 580] g/1.73 m^2^ (*P* = 0.748)). The mean difference in LTM from rest‐to‐post‐exercise was 16.5 [−7.0, 40.0] g/1.73 m^2^ (*P* = 0.489) in the COPD group and 35.2 [11.7, 58.7] g/1.73 m^2^ (*P* = 0.023) in the healthy control group. There was no significant Group × Time interaction. Data on LTM are presented in Figure 2.

**FIGURE 2:**
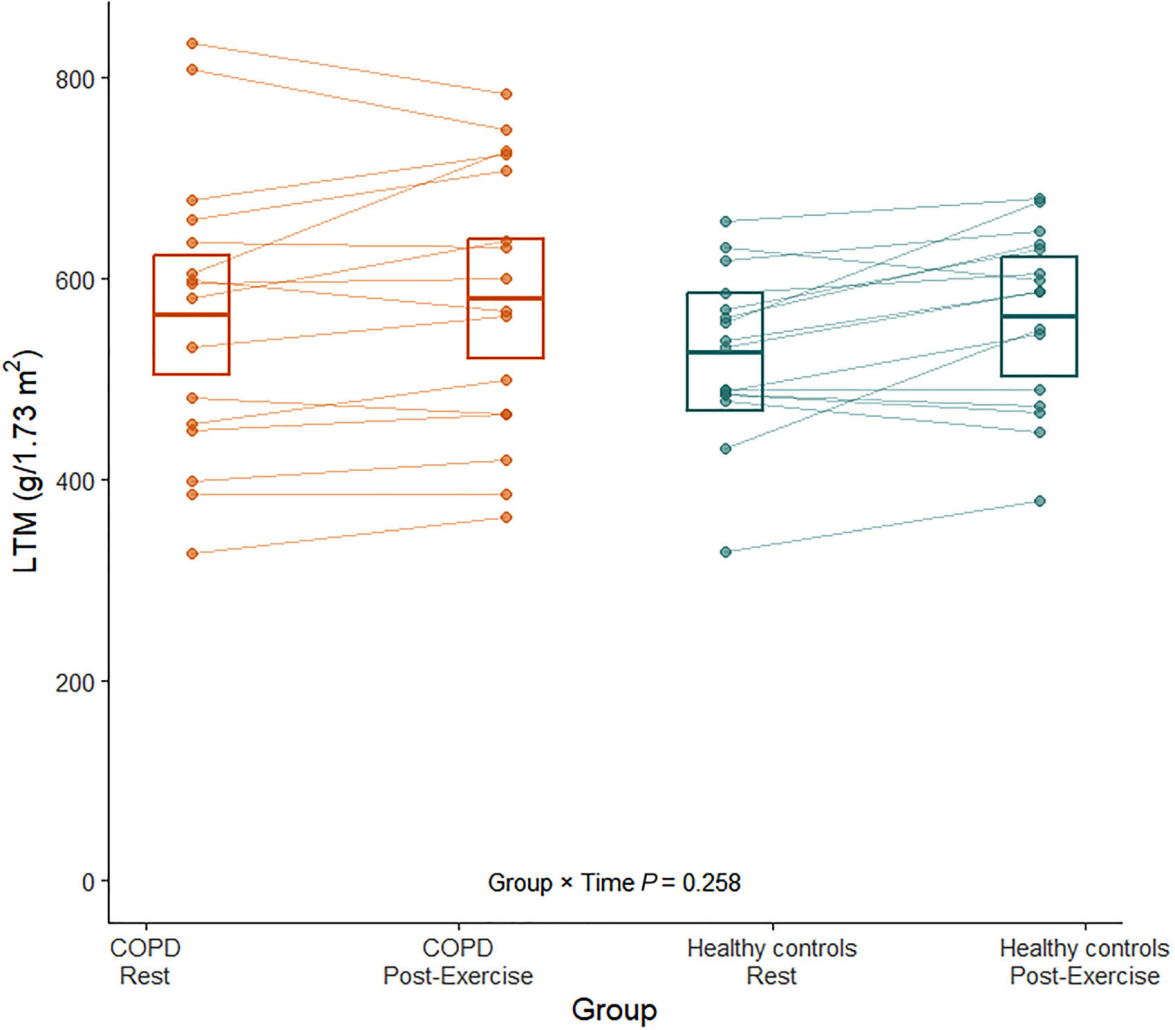
Rest‐to‐post‐exercise changes in lung tissue mass. Note: rest‐to‐post‐exercise changes in lung tissue mass (LTM) normalised to 1.73 m^2^ body surface area in COPD (orange, *n* = 16) and healthy controls (green, *n* = 16). Each dot represents an estimate of a participant's LTM. The boxes show the estimated marginal means (95% confidence interval) averaged over sex. The overall interaction term reflects the Group × Time interaction effect. COPD, chronic obstructive pulmonary disease; LTM, lung tissue mass.

### 3.5 D_L,CO,NO_

The post‐exercise *D*_L,CO,NO_ measurements were performed at the same time points as the LDCT on study day 1. Data on the different *D*_L,CO,NO_ metrics at rest and following exercise are presented in Table 2. *D*_L,NO_ and *D*_M,CO_ increased in both groups from rest‐to‐post‐exercise. Group × Time interaction was not significant for any *D*_L,CO, NO_ metric.

**TABLE 2:** Rest‐to‐post‐exercise changes in pulmonary diffusing capacity.

	COPD			Healthy controls			
	Rest	Post‐exercise	Mean difference	Rest	Post‐exercise	Mean difference	Group × Time interaction, *P*‐value
*D*_L,NO_ (mmol min^−1^ kPa^−1^	24.0 [20.8;27.1]	25.1 [22.0;28.2]	1.14 [0.38;1.90] *P* = 0.023	32.3 [29.2;35.4]	33.8 [30.7;36.9]	1.51 [0.75;2.28] *P* = 0.002	0.484
*D*_L,CO,5s_ (mmol min^−1^ kPa^−1^)	6.0 [5.2;6.7]	6.2 [5.4;6.9]	0.19 [−0.05;0.44] *P* = 0.383	7.6 [6.8;8.4]	8.0 [7.2;8.8]	0.41 [0.16;0.65] *P* = 0.009	0.211
*D*_M,CO_ (mmol min^−1^ kPa^−1^)	18.9 [16.1;21.7]	20.0 [17.2;22.8]	1.11 [0.38;1.85] *P* = 0.021	27.0 [24.2;29.8]	28.1 [25.3;30.9]	1.09 [0.36;1.83] *P* = 0.024	0.962
*V*_C_ (mL)	45.9 [38.6;53.2]	47.8 [40.5;55.1]	1.9 [−0.8;4.6] *P* = 0.481	57.3 [50.0;64.6]	60.9 [53.5;68.2]	3.58 [0.90;6.26] *P* = 0.05	0.372
$eD_{L,O_{2}}$ (mmol min^−1^ kPa^−1^)	12.5 [10.8;14.1]	13.0 [11.4;14.7]	0.56 [0.09;1.03] *P* = 0.091	16.5 [14.9;18.2]	17.4 [15.8;19.1]	0.91 [0.44;1.38] *P* = 0.002	0.295

*Note*: Changes in *D*_L,CO,NO_ metrics for COPD participants (*n* = 16) and healthy controls (*n* = 16) from rest‐to‐post‐maximal‐exercise measured in supine position. Data are presented as estimated marginal mean [95%CI]. The overall interaction term reflects the Group × Time interaction.

Abbreviations: COPD, chronic obstructive pulmonary disease; *D*_L,NO_, pulmonary diffusing capacity for nitric oxide; *D*_L,CO,5s_, pulmonary diffusing capacity for carbon monoxide with 5 s breath‐hold; *D*_M,CO_, alveolar–capillary membrane diffusing capacity for carbon monoxide; $eD_{L,O_{2}}$, estimated pulmonary diffusing capacity for O_2_; *V*_C_, pulmonary capillary blood volume.

## 4 DISCUSSION

The main findings of the present study are that an acute exercise bout performed at maximal exertion did not affect PCI post‐exercise in either individuals with COPD or healthy controls, with no corresponding differences in the associated changes in LTM.

As in previous studies, resting PCI in individuals with COPD was similar to that observed in healthy individuals, thereby differing from findings reported in various other lung diseases, including acute asthma, interstitial lung disease and acute respiratory distress syndrome (Claesson‐Welsh et al., 2021; Coates & O'Brodovich, 1986; Effros et al., 1986; Hanel et al., 2003; Huchon et al., 1984; Rinderknecht et al., 1980). However, in contrast to our working hypothesis, we found no evidence of exercise‐induced alveolar–capillary membrane breaching, that is, no post‐exercise increases in PCI with the results being independent of the degree of static hyperinflation at rest. Accordingly, it is not surprising that no corresponding difference in changes in LTM was observed. If anything, despite the absence of a significant Group × Time interaction at the 0.05 level, one may exploratively observe that an underlying ∼35 g rest‐to‐post‐exercise increase in LTM was observed only in healthy individuals, which cannot be explained by the concomitant increase of approximately 4 mL in *V*_C_ (Table 2) and is therefore consistent with fluid extravasation. While this does not appear to be attributable to altered alveolar–capillary membrane permeability, it may instead relate to the higher workloads and, consequently, higher cardiac outputs achieved by the healthy controls, although this remains speculative, particularly given that the COPD cohort included here was relatively fit and exhibited a comparatively preserved ${\dot{V}}_{O_{2}\mathrm{peak}}$ (Table 1).

Furthermore, as the a priori power calculation was based on PCI rather than LTM, it is also possible that the absence of a significant Group × Time interaction at the 0.05 level for LTM reflects a type II error (Berg et al., 2024). Nonetheless, given that the opposite response was hypothesised, namely an increase in PCI accompanied by a greater increase in LTM in COPD following exercise, the present findings provide no evidence to support exercise‐induced alveolar–capillary membrane breaching with concomitant interstitial fluid accumulation in COPD. In terms of the reported supine *D*_L,CO,NO_ metrics, they were all lower in individuals with COPD than in healthy controls, irrespective of condition, which is consistent with recent *D*_L,CO,NO_‐based findings demonstrating a reduced alveolar–capillary reserve in COPD, as assessed by an upright‐to‐supine postural shift (Rasmussen et al., 2026). Notably, in the present study only *D*_L,NO_ and *D*_M,CO_, with a similar tendency for $eD_{L,O_{2}}$, differed between rest and post‐exercise, showing comparable increases in both COPD and healthy individuals (Table 2). This pattern indicates increased pulmonary capillary recruitment in both groups post‐exercise compared to rest, consistent with the measurements being obtained shortly after the acute exercise bout, at a time when cardiac output would still be markedly elevated. These findings are consistent with pulmonary capillary recruitment being far from maximal in the resting supine position in both COPD and healthy individuals (Madsen et al., 2023; Rasmussen et al., 2026), which is why exercise superimposed on the supine position has also been employed previously to assess alveolar–capillary reserve (Ross et al., 2020). Taken together and given that alveolar–capillary membrane breaching would be expected to result in a reduction rather than an increase in pulmonary diffusing capacity approximately 2 h after exercise (Hanel, 2000), the present findings further contrast with the working hypothesis of exercise‐induced alveolar–capillary membrane breaching in COPD.

Several limitations must be considered. We matched individuals with COPD and healthy controls for age and sex on a 1:1 basis; however, as noted above, the relatively small sample size may have introduced type II errors in the statistical analyses. Subgroup analyses according to disease severity, which might have provided additional insight into alveolar–capillary membrane integrity, were precluded by the limited sample size. Differences in medication use were also not controlled for. Furthermore, participation was likely biased towards the healthiest individuals with COPD, introducing a potential selection bias. Most participants had mild to moderate COPD, with only two participants having severe disease; consequently, our findings may not be generalisable to individuals with more advanced COPD. Because the primary objective was to obtain a valid symptom‐limited maximal CPET, serial IC manoeuvres, the standard method for assessing dynamic hyperinflation during exercise (Guenette et al., 2012), were not performed. Consequently, and although this clearly suggests that dynamic hyperinflation was not prominent in the studied patients, this was assessed only indirectly from the peak *V*_T_/TLC ratio (Chuang, 2023). Also, pulmonary vascular pressures were not measured during exercise. It therefore remains unknown whether these physiological responses were affected to an extent that might have been sufficient to promote pulmonary capillary extravasation. Finally, the PCI results could have been influenced by the 22 min from exercise termination until measurement of PCI in both groups. However, since other studies have found increased PCI 20 and 25 min following exercise, this might have influenced the results to a lesser extent (Hanel et al., 2003; Lorino et al., 1989).

There are also certain caveats associated with PCI as a method for assessing breaching of the alveolar–capillary membrane, because it reflects both epithelial and endothelial permeability. A more ideal approach might have specifically targeted pulmonary capillary permeability. However, even though the epithelial component constitutes the rate limiting step for pulmonary ^99m^Tc‐DTPA clearance, breaching of the pulmonary capillaries would nevertheless be expected to result in an increase in PCI, as documented in several previous studies (Hanel et al., 2003; Lorino et al., 1989; Marks et al., 1985; Meignan et al., 1986), and this method was therefore considered appropriate in the present context. There are, nevertheless, several other technical considerations relevant to the performance and interpretation of the pulmonary ^99m^Tc‐DTPA clearance measurements. The distribution of inhaled ^99m^Tc‐DTPA within the lungs is an important determinant of PCI assessment. If deposition is predominantly central, ^99m^Tc‐DTPA may be partially cleared by mucociliary transport. To minimise central deposition, the inspiratory flow rate should be below 36 L min^−^^1^, particle size below 2 µm, and inspiratory volume slightly deeper than normal, conditions that were all fulfilled in the present study. Accordingly, central deposition was minimal on the scintigraphic recordings. Another aspect requiring consideration is that PCI may be influenced by changes in pulmonary perfusion. This may be relevant here, given that both cardiac output and the distribution of pulmonary perfusion may differ between groups and conditions in the present study. However, pulmonary ^99m^Tc‐DTPA clearance has previously been shown to be minimally affected by alterations in perfusion distribution and/or cardiac output (Rizk et al., 1984), and is therefore probably of lesser practical importance here. The radiochemical purity of ^99m^Tc‐DTPA is another important consideration, as PCI is approximately five times faster for free pertechnetate (^99m^TcO_4_^−^) than for ^99m^Tc‐DTPA. However, in our internal quality control, the in vitro binding of ^99m^Tc‐DTPA was approximately 98%, such that only 1–2% was present as free pertechnetate. Consistent with this, no activity was observed in the thyroid gland or stomach on the scintigraphic recordings, which would have been expected to have a substantial proportion of free pertechnetate present.

In conclusion, the present study found no evidence of alveolar–capillary membrane breaching with concomitant accumulation of interstitial fluid in the lungs, neither in individuals with COPD nor in age‐ and sex‐matched healthy controls following exercise to maximal exertion. Although the present study was strictly mechanistic and dyspnoea per se was not assessed, these findings argue against alveolar–capillary membrane permeability and interstitial fluid accumulation as a significant contributor to persistent post‐exertional dyspnoea in COPD.

## AUTHOR CONTRIBUTIONS

Rie S. Thomsen: Design, data collection, data analysis, data interpretation, figures, first draft, revisions. Iben E. Rasmussen: Data collection, data interpretation, revisions. Stine Buus Nymand: Design, data collection, revisions. Anna Agnes Lytzen: Design, data interpretation, revisions. Milan Mohammad: Data collection, revisions. Jacob P. Hartmann: Design, data collection, data analysis, data interpretation, revisions. Malte Lund Adamsen: Data collection, revisions. Birgitte Hanel: Design,revisions. Jann Mortensen: Design, data analysis, data interpretation, revisions. Ronan M. G. Berg: Conception, design, data interpretation, first draft, revisions, supervision. All authors approved the final version of the manuscript and agreed to be accountable for all aspects of the work in ensuring that questions related to the accuracy or integrity of any part of the work are appropriately investigated and resolved. Ronan M. G. Berg is guarantor of this work and accepts full responsibility for the work and the conduct of the study, had access to the data, and controlled the decision to publish. All persons designated as authors qualify for authorship, and all those who qualify for authorship are listed.

## CONFLICT OF INTEREST

None of the authors have conflict of interest to declare. The authors declare that the research was conducted in the absence of any commercial or financial relationships that could be construed as a potential conflict of interest.

## GENERATIVE AI STATEMENT

During the preparation of this work, ChatGPT (OpenAI, GPT‐5.5) was used for spelling and grammatical correction; no Generative AI contributed to the conceptualisation, analysis, interpretation, or substantive content.

## Figures and Tables

**FIGURE A1:**
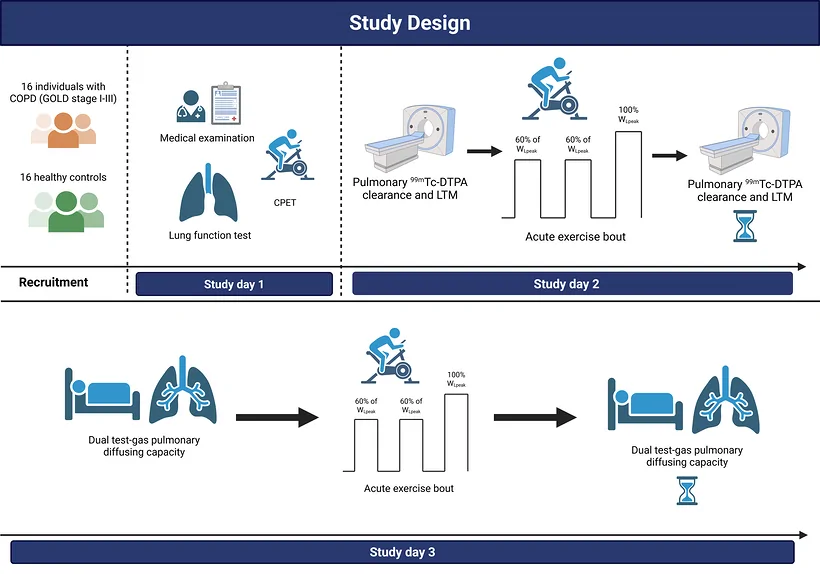
Study design. Note: A total of 16 individuals with COPD and 16 healthy controls underwent three study days with at least 48 h in‐between. Assessment of lung tissue mass (LTM) and dual test‐gas pulmonary diffusing capacity following exercise were executed within the same time frame, in both groups after a minimum of 10 min when the breathing pattern had normalised. Abbreviations: ^99^mTc‐DTPA, ^99^mTc‐labelled diethylenetriaminepentaacetic acid; COPD, chronic obstructive pulmonary disease; CPET, cardiopulmonary exercise test; *W*_Lpeak_, peak workload. Created in BioRender. Berg, R. (2026) https://BioRender.com/it8kdkk.

## Data Availability

The data underlying our findings can be shared upon reasonable request directed to the corresponding author.
